# Dissociating the therapeutic effects of environmental enrichment and exercise in a mouse model of anxiety with cognitive impairment

**DOI:** 10.1038/tp.2016.52

**Published:** 2016-04-26

**Authors:** J Rogers, U Vo, LS Buret, TY Pang, H Meiklejohn, A Zeleznikow-Johnston, L Churilov, M van den Buuse, AJ Hannan, T Renoir

**Affiliations:** 1 Gene Environment & Behaviour Laboratory, Florey Institute of Neuroscience and Mental Health, Melbourne Brain Centre, University of Melbourne, Parkville, VIC, Australia; 2 School of Psychology and Public Health, La Trobe University, Bundoora, VIC, Australia; 3 School of Mathematical and Geospatial Sciences, RMIT University, Melbourne, VIC, Australia; 4 Department of Anatomy and Neuroscience, University of Melbourne, Parkville, Australia

## Abstract

Clinical evidence indicates that serotonin-1A receptor (5-HT_1A_R) gene polymorphisms are associated with anxiety disorders and deficits in cognition. In animal models, exercise (Ex) and environmental enrichment (EE) can change emotionality-related behaviours, as well as enhance some aspects of cognition and hippocampal neurogenesis. We investigated the effects of Ex and EE (which does not include running wheels) on cognition and anxiety-like behaviours in wild-type (WT) and 5-HT_1A_R knock-out (KO) mice. Using an algorithm-based classification of search strategies in the Morris water maze, we report for we believe the first time that Ex increased the odds for mice to select more hippocampal-dependent strategies. In the retention probe test, Ex (but not EE) corrected long-term spatial memory deficits displayed by KO mice. In agreement with these findings, only Ex increased hippocampal cell survival and BDNF protein levels. However, only EE (but not Ex) modified anxiety-like behaviours, demonstrating dissociation between improvements in cognition and innate anxiety. EE enhanced hippocampal cell proliferation in WT mice only, suggesting a crucial role for intact serotonergic signalling in mediating this effect. Together, these results demonstrate differential effects of Ex vs EE in a mouse model of anxiety with cognitive impairment. Overall, the 5-HT_1A_R does not seem to be critical for those behavioural effects to occur. These findings will have implications for our understanding of how Ex and EE enhance experience-dependent plasticity, as well as their differential impacts on anxiety and cognition.

## Introduction

Anxiety disorders are the most common mental illness in the general population (~25% US lifetime prevalence).^1^ The clinical symptoms are often accompanied by cognitive impairment, suggesting that interactions between affective state and cognition may underlie the debilitating nature of pathological anxiety, although little is known in humans regarding the precise nature of either the cognitive deficits or these interactions.^2, 3^ Serotonergic signalling is implicated in the manifestation of various psychiatric disorders and regulates hippocampal-dependent cognitive and emotional processing that can underpin these disorders.^4^ Clinical evidence indicates that functional serotonin-1A receptor (5-HT_1A_R) gene polymorphisms are associated with both anxiety disorders and deficits in cognitive processing.^5, 6^ Constitutive 5-HT_1A_R knock-out (KO) mice have an anxiety-like phenotype, as well as hippocampal-dependent learning and memory deficits.^7, 8, 9, 10, 11^ In addition to prevalent 5-HT_1A_R-targeted drug treatments for anxiety disorders, environmental manipulations such as cognitive-behavioural therapy and exercise (Ex) have already been associated with decreased symptoms of anxiety, as well as improved cognitive functioning in humans.^12, 13^ A meta-analysis of randomized controlled trials demonstrated that Ex elicited greater reductions in anxiety than other forms of anxiety treatment while noting that the mechanism for this effect remains largely unexplained.^14^ In addition, an aerobic Ex regime was found to reduce responses to a high affinity 5-HT_1A_R agonist in patients with an anxiety disorder, indicating that the 5-HT_1A_R may be involved.^15^ In adult rodents, Ex or environmental enrichment (EE) also change emotionality-related behaviours, as well as enhance some aspects of hippocampal-dependent cognition.^16^

The underlying mechanisms mediating the effects of EE and Ex on cognition and anxiety-like behaviour are still unclear. EE is a complex stimulation of sensory, motor and cognitive systems that induces hippocampal-dependent affective and cognitive-behavioural changes in rodents. These changes are correlated with enhanced synaptic plasticity, as well as adult hippocampal neurogenesis and other aspects of experience-dependent cellular plasticity.^17^ The EE literature can be misleading because these protocols often include running wheels as part of the motor stimulation.^18, 19, 20^ This is despite a strong body of evidence having long attributed many of the beneficial effects from EE to just Ex alone.^21, 22, 23^ Very few studies have comprehensively compared the effects of EE (without running wheels) vs Ex. Furthermore, anxiety and cognition have generally been considered separately.^24^ Three recent and unique dissociation studies all confirmed that adult neurogenesis, and mature brain-derived neurotrophic factor (mBDNF), a key potential molecular mediator of synaptic plasticity, were only increased with running wheel access.^25, 26, 27^

Ex raises hippocampal extracellular 5-HT levels, which mediates Ex-induced neurogenesis at the molecular level.^28, 29, 30^ A recent study further elucidated this essential role of 5-HT in Ex-induced neurogenesis, identifying the 5-HT_3_ receptor as critical for the interaction.^31^ We sought to determine whether the 5-HT_1A_R is also crucially involved. Ex increases hippocampal BDNF protein levels^23^ and experience-induced neurogenesis requires BDNF activity.^19, 32, 33^ The serotonergic system and BDNF do interact, for example, antidepressant treatment causing increases in BDNF expression in rodents and humans.^34, 35^ Antidepressants are also known to elevate the rate of neurogenesis and in the 5-HT_1A_R KO this effect does not occur^36^ yet it does seem that this effect is dependent on background strain.^37^ Notably, in a very recent report, through transgenic restoration of dentate gyrus 5-HT_1A_Rs in KO mice on a mixed background, it was firmly established that hippocampal 5-HT_1A_R expression alone is necessary and sufficient for the antidepressant cell proliferation response to occur.^38^ This study demonstrates the requirement of the 5-HT_1A_R in the dentate gyrus for BDNF expression changes in response to fluoxetine, confirming that the interaction discussed above involves the 5-HT_1A_R. However, whether BDNF also mediates the fluoxetine effect on neurogenesis, and if the 5-HT_1A_R is critical to BDNF mediation of Ex-induced neurogenesis, remains unknown.

The impact of anxiety on cognition and the causal relationship between both is still unclear.^24^ We aimed to assess whether Ex or EE would correct behavioural impairments of 5-HT_1A_R KO mice. Through this design, we will also explore the potential role of the 5-HT_1A_R in the molecular and cellular effects of EE and Ex.

## Materials and methods

### Animals and housing

5-HT_1A_R KO mice and WT littermates on a C57BL/6 background were obtained by heterozygous breeding at the Florey Institute of Neuroscience and Mental Health. After weaning, until they were 8 weeks of age, the mice were group-housed (3–5 per cage) by genotype in open-top standard-housed (SH) cages (34 × 16 × 16 cm) with *ad libitum* access to water and food on a 12-hour light/12-hour dark cycle (lights on at 0700 hours). At this point, mice were randomly assigned to EE or Ex groups and were housed in larger cages (40 cm × 28 cm × 18 cm) with access to a variety of objects of different textures, shapes and in differing configurations (changed weekly for novelty) or to running wheels (12 cm diameter), respectively (Supplementary Figure 1A). EE cages did not contain running wheels. Mice of both genotypes that remained in the SH cages described above, designated ‘SH', served as the control condition to which the effects of both treatments were compared. The two genotypes were housed independently from each other; the genotypes were not mixed in any of the housing conditions. At 8 weeks of age there were no significant genotype differences in body weight. We also found no significant genotype or treatment differences in body weight gain for the entire duration of the experimentation (Supplementary Figure 1B). Finally, there were no significant genotype differences in the daily distance run after 5 weeks of access to running wheels (Supplementary Figure 1C).

### Behavioural testing

Behavioural experiments were performed at different time points, with anxiety testing at 10 weeks of age and long-term spatial memory testing beginning at 12 weeks of age (Supplementary Figure 1A). These time points correspond to 2 weeks and 4 weeks of treatment, respectively, with all tissue collected a few days after the termination of behavioural testing (that is, 13 weeks of age) for the molecular and cellular analyses described below. Mice were inspected daily for any signs of distress. No signs of morbidity (including seizures) have been observed throughout the whole study. All experiments were performed blind to genotype (and treatments when possible) in accordance with the guidelines of the Florey Institute's Animal Ethics Committee and the National Health and Medical Research Council (NHMRC). All parameters were measured using TopScan v 2.0 tracking software (Clever Sys, Restin, VA, USA).

### Elevated plus maze

We assessed anxiety-like behaviour using the elevated-plus maze (EPM). The EPM comprised of two open arms (36 × 5 cm) and two closed arms (36 × 5 × 18 cm) that extended from a central platform (5 × 5 cm). The entire apparatus was elevated 50 cm from the floor and made of beige Perspex. The time spent in the arms and the central platform, as well as the total distance moved was recorded. The test duration was for 5 min.

### Morris water maze

The reference version of the Morris water maze was designed according to the study by Vorhees and Williams.^39^ A circular 1.2 m pool was filled with water at a temperature of 22±2 °C that was made opaque with nontoxic white paint to conceal a 10 cm diameter platform placed 0.5 cm below the water surface. The pool was placed in a room with numerous high-contrast 2D cues located distally on the room walls and on a curtain hiding the experimenter and recording equipment, as well as 3D cues including the heating lamps and holding containers, to provide multiple landmarks within the room for the mice to use to navigate and learn the location of the hidden platform. All mice, regardless of genotype or treatment, were trained to navigate to the exact same spatial location, that is, the hidden platform location remained constant throughout experimentation.

### Spatial learning assessment

To assess spatial learning, mice were trained to use the cues surrounding the pool to form a spatial map of the arena and thus acquire the hidden platform location. Each mouse was trained for 6 days with four trials per day, from four distinct start positions, with the order of starting locations randomised on each day. Mice were allowed to search for the platform location for 60 s and if they did not find its location they were gently guided to it. After finding the platform, mice were left on it for 30 s, to promote spatial mapping of its location. Subsequently, they were removed and placed in holding containers underneath heating lamps for ~20 min before their next trial (mice were trained sequentially in sessions of 12–14 mice, the inter-trial interval was the time taken for all the other mice to perform their trials).

### Spatial memory assessment

A 24-hour retention probe with the hidden platform removed from the pool was performed after the last day of training to assess long-term spatial memory. The trial began with the mice starting from a novel position directly across from the hidden platform location and lasted for 2 min. The mouse behaviour was analysed in blocks of 30 s. Long-term spatial memory formation, the specific memory engram for the platform location, was determined by preference for the target quadrant.^39^ Target quadrant preference was defined as mice spending statistically significant more time in the quadrant containing the hidden platform during spatial learning compared with chance (that is, 25% of time analysed). Annulus crossings were recorded during the probe trial as bouts in the exact former location of the hidden platform, determined by the centre of the mouse entering that location. To quantify accuracy for the former platform location, the annulus crossing index was derived from annulus crossings (defined as above) of the relative platform position in each of the other quadrants as per the study by Janus.^40^

### Morris water maze learning analysis

In addition to assessing general spatial learning with classical parameters such as latency to platform or path length, we performed a search strategy analysis using time-tagged *xy*-coordinates derived from Topscan using a algorithm in Matlab (Mathworks, Natick, MA, USA) as previously described.^41^ Briefly, the strategy mice employ during each trial to find the hidden platform was quantified into one of seven strategies, ranging from less hippocampus-dependent, unspatial or ‘egocentric', to more hippocampus-dependent, spatial or ‘allocentric' in nature.^42^ The strategies were dichotomised as egocentric or allocentric and this classification was used as input for statistical analysis. The % spatial search strategy was used as an indicator of allocentric hippocampal-dependent spatial learning. Treatment effect sizes were estimated as odds ratios (ORs) with corresponding 95% confidence intervals (95%CIs) to quantify the precision of the estimated effects.

### DOI-induced head-twitches

An additional cohort of mice was used to assess the effect of EE on DOI-induced head-twitches. At 12 weeks of age (that is, after 4 weeks of EE), all the mice were administered with the 5-HT_2_ receptor agonist DOI (0.3 mg kg^−1^, i.p.) and immediately placed inside an observation area. The number of head-twitches was counted for a 15-min period starting 15 min after drug administration.

### Adult-born hippocampal cell survival and proliferation

5-bromodeoxyuridine (BrdU, Sigma-Aldrich, Castle Hill, NSW, Australia) was dissolved in 0.9% saline and injected i.p. at a dose of 50 mg kg^−1^ of body weight for 7 consecutive days (when mice were 8 weeks of age) at the beginning of EE or Ex.

### Tissue processing

Five weeks after the first injection of BrdU, and a few days after the end of behavioural analysis, all animals were killed and intracardially perfused (see Supplementary Figure 1A). This time point corresponds to ~5 weeks of exposure to both Ex and EE. Brain tissue was obtained and stored at −80 °C for immunohistochemistry as previously described.^43^ Serial coronal hippocampal sections from Bregma coordinates −1.34 to −2.54 mm were cut on a cryostat (Leica CM1900, Leica Biosystems Melbourne, Mount Waverly, VIC, Australia) at 40-μm thickness, were collected in a 1 in 6 series spaced 240 μm apart, and immediately placed in a cryopreserve solution of 0.1 m Phosphate buffer containing 25% v/v ethylene glycol (VWR International, Leuven, Belgium) and 25% v/v (Chem-Supply, Gilman, SA, Australia). Collected series were then stored at −20 °C until used for peroxidase immunohistochemistry.

### Peroxidase BrdU and Ki67 immunohistochemistry

Peroxidase immunohistochemistry was performed essentially as described previously.^43^ Briefly, floating sections were incubated in hydrogen peroxide (1% in PBS) to destroy endogenous peroxidase activity; underwent antigen unmasking in 1 m HCL for 35 min at 37 °C (for BrdU only); were incubated in blocking buffer consisting of 0.2% Triton X (Sigma-Aldrich) and 5% v/v normal donkey serum (Merck-Millipore, Kilsyth, VIC, Australia) in 100 mm PBS for 1 h; were incubated in either sheep anti-BrdU (Exalpha Biologicals, Watertown, MA, USA) diluted 1/1000 or rabbit anti-Ki67 (Thermo Fisher Scientific Australia, Scoresby, VIC, Australia) diluted 1/200 in blocking buffer overnight at 20 °C; were incubated with biotinylated rabbit anti-sheep (Vector Laboratories, Burlingame, CA, USA) or biotinylated goat anti-rabbit Ki67 (Vector Laboratories) diluted at 1/500 for 2 h; incubated with Vectastain ABC solution (1:100, Vector Laboratories) for 1 h; developed with diaminobenzidine liquid chromogen kit (1:50, Dako Australia, North Sydney, NSW, Australia); were mounted on slides and then cover-slipped with DPX. Unless otherwise stated, each step occurred at room temperature and was followed by thorough washing with 100 mm PBS.

### BrdU+ and Ki67+ cell counts

Slides were coded and the identity of the treatment and genotype was blinded to the observer who performed the counting. For each animal, the total numbers of dentate gyrus BrdU+ and Ki67+ cells were counted within the granule cell layer (GCL) or subgranular zone (SGZ) respectively, using an UPlanFL N × 60 objective lens and an Olympus BX61 light microscope (Olympus America, Center Valley, PA, USA). In each section, digital images were captured using a × 10 objective lens and a Micropublisher 5.0 RTV camera (Q Imaging, Surrey, BC, Canada), while Image Pro Plus 6.0 software (Media Cybernetics, Rockville, MD, USA) was used to measure the GCL surface area and SGZ length of both the suprapyramidal and infrapyramidal blades of the dentate gyrus. These values were converted to volumes in μm^3^ by multiplying by 40 μm to give the volume of the GCL, or 40 × 32 μm for the SGZ volume. These values were converted to mm^3^ and then divided into the number of BrdU+ or Ki67+ cells to give a density in cells per mm^3^. Results were expressed by averaging the density from all sections, and expressed as BrdU+ or Ki67+ cells per mm^3^±s.e.m. BrdU+ cells per mm^3^ were used to quantify the effect of our various manipulations on adult-born cell survival; Ki67+ cells per mm^3^ were used to quantify the chronic effect (that is, after 5 weeks of Ex or EE) of our various manipulations on adult-born cell proliferation. Both measures were quantified to measure aspects of dentate gyrus cellular plasticity that are known surrogates of adult neurogenesis in the hippocampus.

### BDNF protein levels

#### Antibodies and reagents

Primary antibodies used were: anti-mature BDNF (mBDNF, H-117,SC20981, Santa Cruz Biotechnology, Santa Cruz, CA, USA), anti-proBDNF (EPR1292, ab108383, Abcam, Melbourne, VIC, Australia) and anti-βactin (A5316, Sigma-Aldrich). Anti-mouse or anti-rabbit horseradish peroxidase-linked secondary antibodies were from Cell Signaling Technology (Danvers, MA, USA). The bicinchoninic acid (BCA) protein assay kit was from Thermo Scientific (Rockford, IL, USA). Detection systems used were LumiGLO Reagent (Cell Signalling Technology, Danvers, MA, USA) and Western Lightning Ultra (PerkinElmer, Waltham, MA, USA).

#### Western blot analysis

Tissue samples were weighed and lysed with 100 μl/0.01 g RIPA buffer (50 mm Tris pH 8.0, 0.1% SDS, 1% Triton X-100, 150 mm sodium chloride, dH_2_O, phosphatase inhibitor (1:50) and protease inhibitor (1:200)) and maintained on ice. Samples were then sonicated, left on a rotator for 45 min and centrifuged for 18 min at 13 000 *g* at 4 °C to remove debris. Protein concentrations were determined by the BCA method. Volumes containing 50 μg of protein were mixed with an equal volume of loading buffer (0.4 m Tris pH 6.8, 37.5% glycerol, 10% SDS, 1% 2-mercaptoethanol, 0.5% bromphenol blue, dH_2_O) and then denatured at 95 °C. Samples were resolved by SDS–polyacrylamide gel, (4–15% Mini-PROTEAN TGX precast polyacrylamide gels (BIORAD, Regents Park, NSW, Australia), or 8% acrylamide gels) at 120 V. The proteins were transferred onto nitrocellulose membranes: overnight at 30 V and 4 °C then 1 h at 30 V the next morning. Membranes were blocked for 2 h at room temperature for proBDNF, or overnight at 4 °C for mBDNF in TBST (20 mm Tris, 150 mm sodium chloride, 0.1% TWEEN 20, dH_2_O) with 5% non-fat milk. Primary antibodies, anti-mBDNF (1:200), anti-proBDNF (1:2000), anti-βactin (1:10 000), were incubated in TBST with 5% BSA overnight at 4 °C. After 1.5-h incubation at room temperature with either anti-mouse or anti-rabbit horseradish peroxidase-linked secondary antibodies, images were captured using a Luminescence Image Analyzer (LAS-4000; FujiFilm Life Science, Stamford, CT, USA) and analysed using Image Quant software (GE Healthcare, Baulkham Hills, NSW, Australia).

### qPCR for assessment of mRNA expression

Quantitative PCR (qPCR) was performed following MIQE guidelines as previously described.^44^ Briefly, total RNA was extracted using an RNeasy Mini Kit (Qiagen, Melbourne, VIC, Australia) and was adjusted to 1000 ng μl ^−1^ for conversion to complementary DNA (cDNA) by reverse transcription using a Superscript Vilo cDNA synthesis kit (Invitrogen, Melbourne, VIC, Australia; Life Technologies Australia, Mulgrave, VIC, Australia). The following primers were used: cyclophilin F 5′-CCCACCGTGTTCTTCGACA-3′ R 5′-CCAGTGCTCAGAGCTCGAAA-3′ 5-HT_3_ F 5′-CATGTATGCCATCCTCAACG-3′ R 5′-GGGATGGACAATTTGGTGAC-3′; 5-HT_1B_ F 5′-AGTCCTGCTGGTTGCTTTGT-3′ R 5′-ATCAGGTAGTTAGCCGGGGT-3′; 5-HTT F 5′-CTTCAGCCCCGGATGGTT-3′ R 5′-GTGGACTCATCAAAAAACTG;CAAA-3′; 5-HT_2C_ F 5′-TGCCATCGTTTGGGCAATA-3′ R 5′-CGTCCCTCAGTCCAATCACA-3′ 5-HT_7_ F 5′-AGTGCCAGTACCGGAATATCAAC-3′ R 5′-CCGCTCTGGATCATGTATCATG-3′; 5-HT_2A_ F 5′-GAACCCCATTCACCATAGCCG-3′ R 5′-CGAAGACTGGGATTGGCATGG-3′. Using the ViiA7 Real Time PCR system (Applied Biosystems, Foster City, CA, USA) and SYBR Green Jumpstart Taq Ready Mix (Sigma-Aldrich, St Louis, MO, USA) qPCR was performed for each target gene for 40 cycles with the following parameters: 95 °C for 15 s then 60 °C for 60 s. The expression of each target gene was determined relative to the reference gene Cyclophilin using the ^ΔΔ^*C*_q_ method.

### Statistics

**Figure 1:**
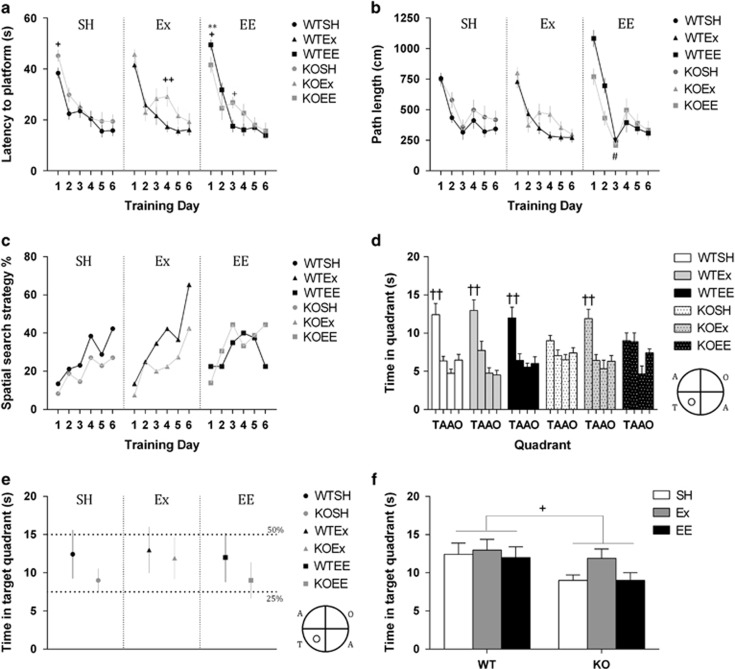
Exercise (Ex) but not environmental enrichment (EE) rescues Morris water maze retention probe performance in 5-HT_1A_ receptor knock-out (KO) mice. (**a**) Latency to the platform during spatial acquisition training over the 6 training days shows no differences in the ability of any mice to acquire the location of the hidden platform by day 5 or 6. (**b**) Mirroring the latency to platform results, the path length the mice chose to find the platform was also unaltered by day 5 or 6. (**c**) Search strategy analysis revealed a clear effect of learning, with the odds of animals choosing spatial strategies increasing across training days (OR 1.31, 95%CI 1.21–1.41, *P*<0.001). It also revealed a KO impairment in the OR to choose more spatial strategies (OR 0.70, 95%CI 0.5–0.99, *P*<0.05) and it also demonstrated that Ex (OR 1.73, 95%CI 1.06–2.83; *P*<0.05) but not EE (OR 1.07; 95%CI 0.63–1.79; *P*>0.05) caused a significant increase in the odds to adopt a spatial strategy. (**d**) Ex restores quadrant preference in the 5-HT_1A_R KO mice during the first 30 s of the retention probe. Preference for the target quadrant (T) for each group was defined by the time spent in target being significantly different to all the remaining quadrants (A, adjacent quadrant; O, opposite quadrant). (**e**) During the retention probe, inferring whether the 95%CIs of estimates of the amount of time spent in a target quadrant cross the quarter-time line (7.5 s) also demonstrates that Ex restores quadrant preference in 5-HT_1A_R KO mice. In 95% of cases, time in the target quadrant was not equal to chance and thus all WT mice exhibited quadrant preference. Standard-housed 5-HT_1A_R KO mice did not show a preference for the target quadrant as the 95%CI overlapped with chance, and thus had impaired long-term spatial memory. Enriched 5-HT_1A_R KO also did not show quadrant preference, but exercising KOs had the memory impairment rescued as shown by a quadrant preference comparable to WT mice. (**f**) Analysis of just time in target quadrant revealed no interaction as suggested from the quadrant preference data (F_2,60_=0.61, *P*>0.05). It did reveal a significant effect of genotype (F_1,60_=5.59, ^+^*P*<0.05) but not of treatment (F_2,60_=1.40, *P*>0.05). Data are expressed as mean±s.e.m., with the exceptions of the search strategy data (expressed as a percentage of spatial strategies selected within all trials on a given day) and the quadrant preference point estimates (expressed as mean±CI); *n*=9–13; Bonferroni pair-wise comparisons: WTEE vs WTSH, **P<0.01; genotype (within treatment condition): ^+^*P*<0.05; ^++^*P*<0.01; quadrant (T vs A, T vs A, & T vs O): ^††^*P*<0.01. CI, confidence interval; OR, odds ratio; SH, standard housing; WT, wild type.

All data, except for the MWM search strategy analysis and quadrant preference determination, are expressed as mean±s.e.m. SH was considered the control condition and EE and Ex were considered as treatments for all statistical analysis. Using SPSS Statistics software (IBM Australia, St Leonid's, NSW, Australia), data were analysed by two-way analysis of variance (ANOVA) with repeated measures where appropriate (with days as repeated measures factors for MWM spatial learning parameters and MWM cued learning), for all analysis except that of search strategy selection and the determination of quadrant preference on the Morris water maze. Between-group factors were genotype and treatment. Dunnet's test pair-wise comparisons were conducted when two-way ANOVA main effects or interactions were significant. For main effects of treatment, Dunnet's test pair-wise comparisons determined if the overall effect was due to Ex or EE. When two-way repeated-measures analyses were used, Bonferroni pair-wise comparisons were conducted when main effects or interactions were significant. Significance threshold was set at *P*<0.05 for all analysis. For clarity, a complete compilation of all ANOVA analysis for the entire study has been provided (see Supplementary Tables 1&2). For the determination of long-term spatial memory formation, quadrant preference is historically defined as the time spent in the target quadrant being significantly different to time spent in each of the other quadrants through ANOVA analysis (see Figure 1d). However, knowledge of time spent in three of the quadrants automatically determines the time in the final quadrant so these measures are not strictly independent from each other and ANOVA analysis is not appropriate despite its prevalence in the MWM literature.^10, 22, 45^ Thus, to be comparable with the existing literature on the 5-HT_1A_R KO, we report the ANOVA analysis (Figure 1d), but we also chose a novel approach of determining quadrant preference. This involved using point estimates with the uncertainty of that estimate (that is, mean+95%CI) for each group to test the null hypothesis that the time spent in the target quadrant was different to chance (that is, 25% of time during probe). A group was determined to possess quadrant preference, and thus intact spatial memory formation, only if the 95%CI of the point estimate did not overlap with chance (see Figure 1e). For the search strategy analysis, a random-effects logistic regression model with individual animals treated as random effects, was used to investigate the association between the search strategy (dichotomised into allocentric vs egocentric strategies),^46^ genotype, treatment and day, adjusting for experimental cohort and start location. Sample size were chosen based on power calculations (*α*=0.05) to detect effect sizes as small as 0.15.

## Results

### Ex, but not EE, rescues the learning and memory impairment displayed by 5-HT_1A_R KO mice

We assessed the effect of Ex and EE on learning and memory using the Morris water maze (Figure 1; Supplementary Figure 2). We controlled for the ability of animals in both genotypes to learn to swim to a cued goal and found no significant differences between them or any effect of EE or Ex (Supplementary Figure 2A). During the spatial learning phase of the task, all groups improved their performance by decreasing their average latency to the platform as the training days progressed (Figure 1a; F_5,280_=88.3, *P*<0.001). Along with an overall effect of genotype (F_1,56_=5.65, *P*<0.05), we also found a significant day × treatment × genotype interaction (F_10,280_=2.14, *P*<0.05), indicating subtle differences between the groups in the acquisition of the platform location. There were genotype differences in each housing condition within the first 4 days of training: increased latencies of 5-HT_1A_R KO compared with WT on day 1 in SH mice (*P*<0.05) and in Ex mice on day 4 (*P*<0.01). In contrast to day 1 SH mice, WTEE mice had increased latencies (*P*<0.01) compared with KO mice, but despite this KO EE mice had increased latencies compared with WT on day 3 (*P*<0.05). Reflecting these day 1 results in EE and SH mice, enriched WT mice had significantly longer latencies to reach the platform than SH controls on the first day of training (*P*<0.05). Importantly, however, there were no significant differences between any groups on either day 5 or 6 of training, demonstrating that all mice, regardless of genotype or housing conditions, had acquired the location of the hidden platform.

Analysing the path lengths throughout training (Figure 1b), we found a significant effect of day (F_5,280_=70.04, *P*<0.001), further demonstrating spatial learning during experimentation through reduction of the path length taken to find the platform location. We also revealed significant day × genotype (F_5,280_=2.95, *P*<0.05), day × treatment (F_5,280_=5.73, *P*<0.001) and genotype × treatment (F_5,56_=4.4, *P*<0.05) interactions. Post hoc tests showed that enriched animals had longer path lengths on the first day of training (*P*<0.05) and WT animals had shorter path lengths than 5-HT_1A_R KO animals on day 4 (*P*<0.05). On the third day of training, EE mice and Ex mice had shorter and longer path lengths compared with SH (*P*<0.05), respectively. Further establishing that all mice, regardless of genotype or environment, had learned the hidden platform location, there were no significant differences in the path length the mice chose on either day 5 or 6 of training. We also report differences in the velocities of treated animals that do not manifest as differences in the latency or path length described above but no effect of genotype (Supplementary Figure 2B).

Our analysis of search strategy confirmed that hippocampal-dependent learning was taking place in all mice (Figure 1c). Assuming similar genotype and treatment, there was a clear learning effect as the odds of adopting an allocentric strategy increased by ~30% per day (day: OR 1.31, 95%CI 1.21–1.41, *P*<0.001). However, we also identified significant differences between WT and KO mice. Assuming similar treatment and day, the 5-HT_1A_R KO mice were 30% less likely to choose an allocentric strategy (genotype: OR 0.70, 95%CI 0.5–0.99, *P*<0.05). We also found differing effects of housing condition, as, assuming similar genotype and day, there was a significant increase in the odds to adopt an allocentric strategy between SH and Ex (OR 1.73, 95%CI 1.06–2.83; *P*<0.05) but not SH and EE (OR 1.07; 95%CI 0.63–1.79; *P*=0.81).

Analysing the first 30 s of the retention probe test (in which the platform was removed from the pool, Figures 1d and e), we found that WT animals displayed preference for the target quadrant compared with the other quadrants regardless of housing conditions (SH12.42 s, 95%CI 9.23–15.61; Ex 12.98 s, 95%CI 9.95–16.01; EE 11.98 s 95%CI 8.78–15.19). In contrast, 5-HT_1A_R KO SH mice did not show any preference for the target quadrant (9 s, 95%CI 7.44–10.56), as the uncertainty of the point estimate overlapped with that of chance (7.5 s), suggesting impairment in long-term spatial memory (Figure 1e). Interestingly, this deficit was no longer observed in the 5-HT_1A_R KO Ex mice (11.92 s, 95%CI 9.20–14.64). However, 5-HT_1A_R KO EE animals still did not exhibit intact memory (9 s, 95%CI 6.64–11.37). These results are in complete agreement with assessing quadrant preference using traditional ANOVA analysis (Figure 1d). WT animals and exercising KO animals demonstrate quadrant preference (*P*<0.05), while SH and enriched KO animals did not statistically spend more time in the target quadrant compared with the other three existing quadrants (*P*>0.05). We then compared time in target between all the groups (Figure 1f) but found no interaction as suggested from the quadrant preference data (F_2,60_=0.61, *P*>0.05). We did find a significant effect of genotype (F_1,60_=5.59, *P*<0.05) but not of treatment (F_2,60_=1.40, *P*>0.05). There were no significant differences in the latency to find the old platform location, the number of times the mice frequented that location or the relative accuracy of their visits (as measured by the annulus crossing index; Supplementary Figures 2C–E).

### EE but not Ex reduces anxiety-like behaviour

**Figure 2:**
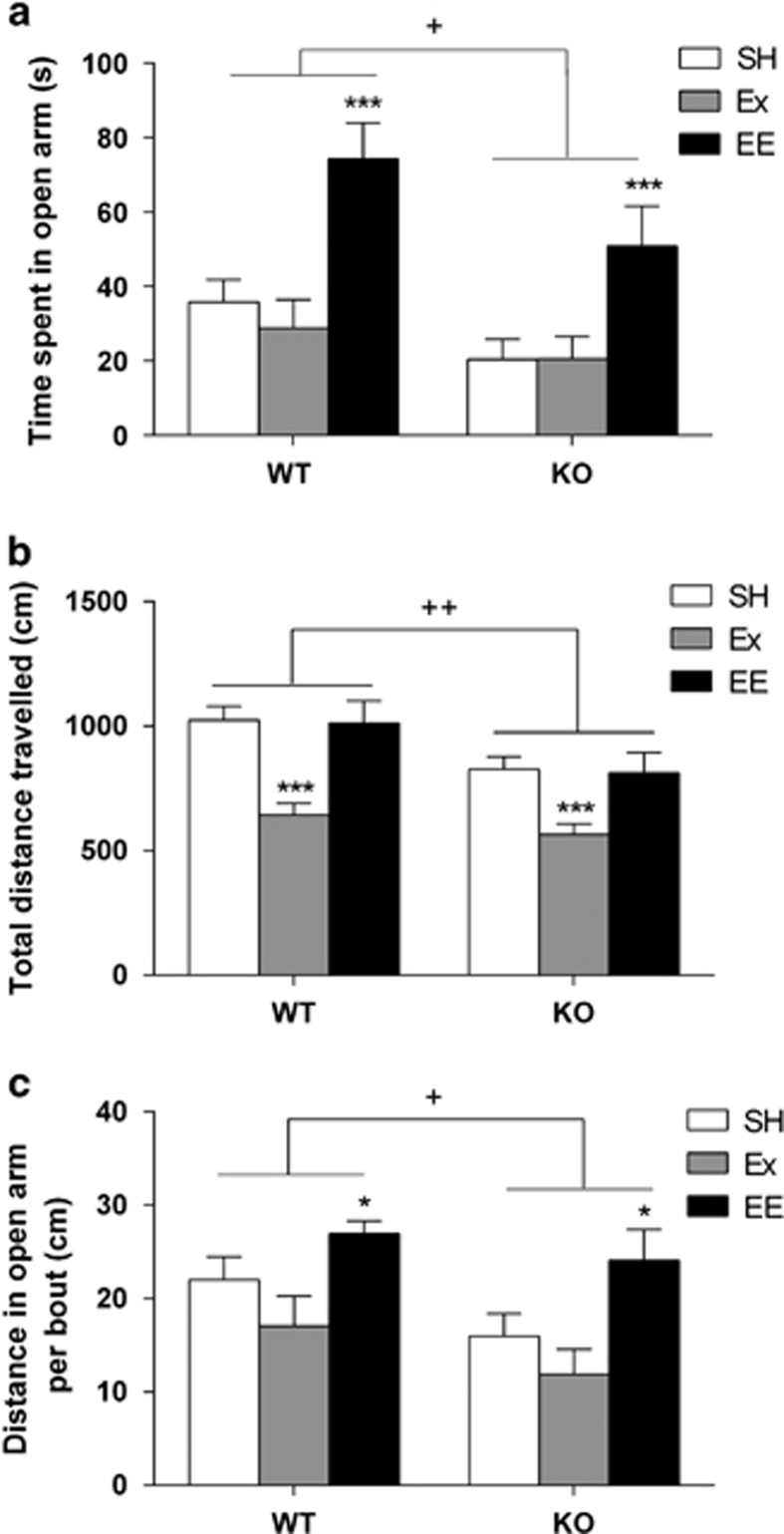
Environmental enrichment (EE) decreases anxiety-like behaviour in the elevated-plus maze (EPM). (**a**) 5-HT_1A_R KO mice spent less time in the open arm than WT littermates, demonstrating anxiety-like behaviour (F_1,110_=6.28, ^+^*P*<0.05). EE but not exercise (Ex) significantly increases the amount of time mice of both genotypes spend in the open arm (F_2,110_=13.6, *P*<0.001; SH vs EE, ***P<0.001); the 5-HT_1A_R KO SH impairment is restored beyond the WTSH level. (**b**) 5-HT_1A_R KO mice move less than WT animals during the test period (F_1,110_=9.325, ^++^*P*<0.01). Ex also significantly decreases the distance travelled in all animals (F_2,110_=16.428, *P*<0.001; SH vs Ex, ***P<0.001). (**c**) 5-HT_1A_R KO mice travel less per bout to the open arm (F_1,110_=4.62, ^+^*P*<0.05). EE increases the distance travelled per open arm bout but there is no effect of Ex (F_2,110_=7.89, *P*<0.001; SH vs EE, *P<0.05). Data is expressed as mean±s.e.m.; *n*=13–25.

In the EPM (Figure 2a), 5-HT_1A_R KO animals showed anxiety-like behaviour, spending less time in the open arms than WT mice (F_1,110_=6.28, *P*<0.05). In addition, EE increased time spent in the open arms, regardless of genotype ( F_2,110_=13.6, *P*<0.001; SH vs EE, *P*<0.001). There was, however, no treatment by genotype interaction for time spent in the open arms (F_2,110_=0.45, *P*>0.05). We also observed significant effects of genotype (F_1,110_=9.325, *P*<0.01) and treatment (F_2,110_=16.428, *P*<0.001) on total distance travelled (Figure 2b). Overall, exercising animals travelled less during the test (SH vs Ex, *P*<0.001). Finally, looking at the ratio of total distance explored over the number of bouts in the open arm (Figure 2c), we found an effect of genotype (F_1,110_=4.62, *P*<0.05) and enrichment (F_2,110_=7.89, *P*<0.001; SH vs EE, *P*<0.05).

### Ex, but not EE, increases adult-born cell survival

**Figure 3:**
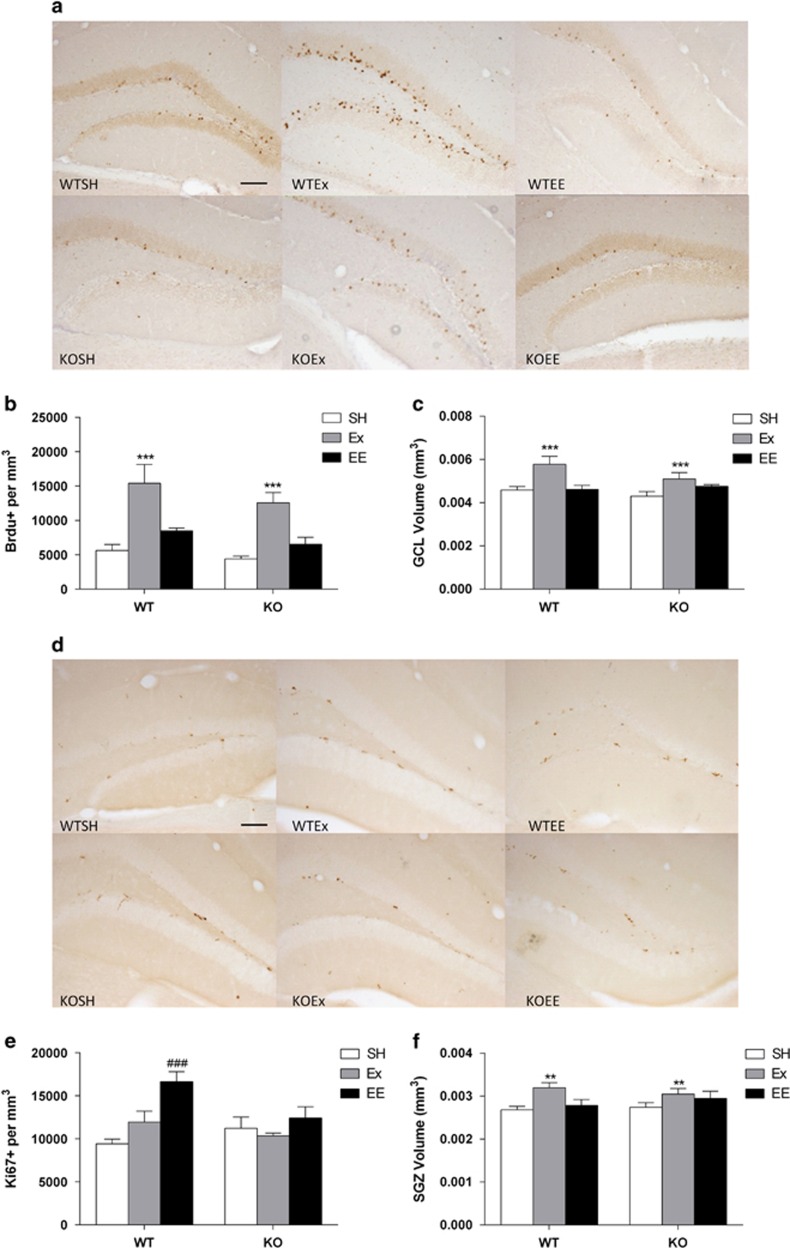
Exercise (Ex) not environmental enrichment (EE) increases the rate of adult-born hippocampal cell survival. Chronic EE but not Ex increases the rate of proliferation as measured by the mitotic marker Ki67 in WT animals only (**a**) Representative micrographs of DAB staining BrdU+ cells in the dentate gyrus granule cell layer (GCL) of both genotypes for each treatment condition. (**b**) The total adult-born cell survival counts from both blades of the dentate gyrus is significantly increased in both WT and 5-HT_1A_R KO mice with access to exercise (F_2,33_=28.27, *P*<0.001; SH vs Ex, ***P<0.001). (**c**) The GCL volume is increased in exercising mice of both genotypes (F_2,33_=9.66, *P*<0.001; SH vs Ex, ***P<0.001). (**d**) Representative micrographs of DAB stained Ki67+ cells in the dentate gyrus sub granular zone (SGZ) of both genotypes for each treatment condition. (**e**) The total proliferation counts from both blades of the dentate gyrus are significantly increased in WT enriched mice but not KO enriched mice (F_2,29_=3.99, *P*<0.05; WTSH vs WTEE, ^###^*P*<0.001). (**f**) The volume of the SGZ is increased in exercising mice but not enriched mice (F_2,29_=5.962, *P*<0.01; SH vs Ex, **P<0.05). Data is expressed as mean±s.e.m.; *n*=5–9. KO, knock out; SH, standard housing; WT, wild type.

We assessed the rate of adult-born cell survival per dentate gyrus (Figure 3). There was a main effect of treatment on adult-born survival per dentate gyrus (Figure 3b; F_2,33_=28.27, *P*<0.001). The number of BrdU+ cells per mm^3^ was dramatically increased in Ex (*P*<0.001) but not EE animals despite a trend for significance (*P*=0.052). Additionally, there was a trend for a genotype effect as 5-HT_1A_R KO animals tended to have lower levels of adult-born cells (F_1,33_=3.83, *P*=0.059). There was also a main effect of treatment on the volume of the granule cell layer (GCL) (Figure 3c; F_2,33_=9.66, *P*<0.001). Ex increased GCL volume compared with SH controls (*P*<0.001). Segregating sections along the dorsal–ventral axis revealed no differences from the above analysis except the trend towards a significant genotype effect became significant (*P*<0.05) in the dorsal hippocampus (Supplementary Figure 3).

### EE, but not Ex, increases chronic proliferation levels

We also assessed the rate of progenitor cell replication using the mitotic marker, Ki67 (Figure 3). In contrast to the BrdU results, we found a genotype by treatment interaction with the Ki67+ cells per mm^3^ in the dentate gyrus (Figure 3e; F_2,29_=3.99, *P*<0.05). The 5-HT_1A_ receptor was critical to this effect as Ki67+ cells per mm^3^ were increased in WT mice only (WTSH vs WTEE, *P*<0.001). We also observed an effect of treatment (F_2,29_=7.98, *P*<0.01), where overall EE had more Ki67+ cells per mm^3^ (*P*<0.01). There was a main effect of treatment on the volume of the SGZ (Figure 3f; F_2,29_=5.962, *P*<0.01). Surprisingly, SGZ volume was increased in Ex but not EE animals (*P*<0.01).

### Ex, but not EE, increases hippocampal BDNF levels

**Figure 4:**
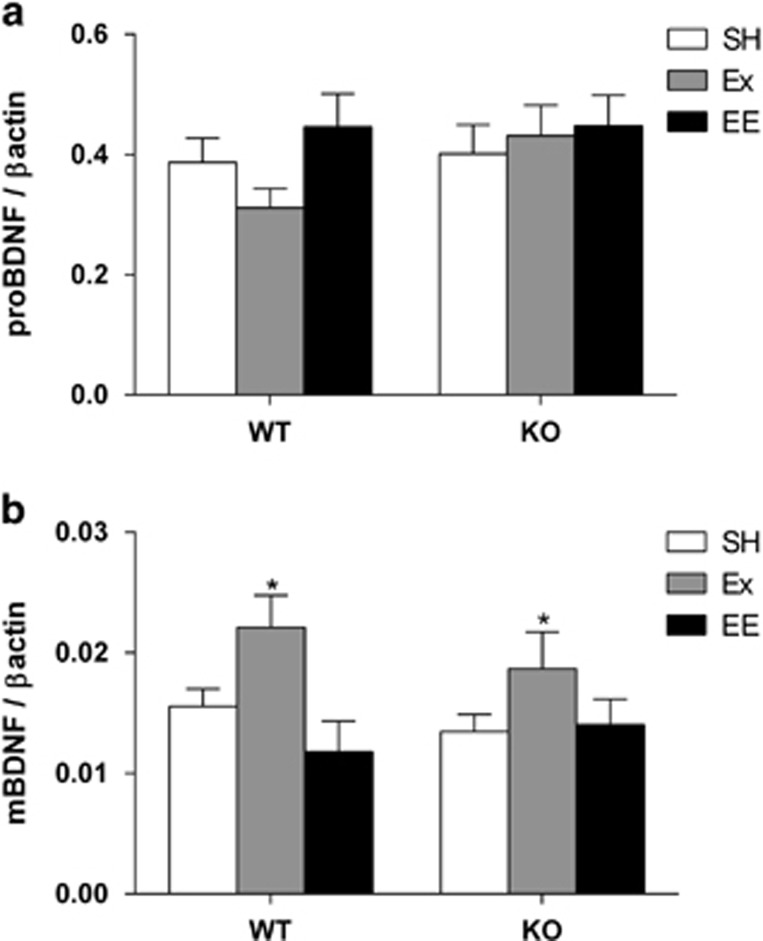
Exercise (Ex) increases levels of hippocampal mature brain-derived neurotrophic factor (mBDNF) in both WT and KO mice. (**a**) There are no significant differences in the proBDNF levels in any groups. (**b**) The total amount of mBDNF protein is significantly increased in both WT and 5-HT_1A_R KO mice with access to Ex (F_2,36_=5.91, *P*<0.01; SH vs Ex, *P<0.05). Data are expressed as mean±s.e.m.; *n*=6–8. KO, knock out; SH, standard housing; WT, wild type.

The levels of hippocampal BDNF protein were assessed by western blot (Figure 4). There were no differences between the groups in hippocampal proBDNF protein levels (Figure 4a; genotype: F_1,42_=1.361, *P*>0.05; treatment: F_2,42_=1.246, *P*>0.05). In contrast, we found a significant effect of treatment (F_2,36_=5.91, *P*<0.01) on mBDNF levels (Figure 4b). Dunnett's pair-wise comparisons revealed that only Ex increased mBDNF levels (*P*<0.05).

### EE, but not Ex, increases hippocampal 5-HT_2C_ gene expression

**Figure 5:**
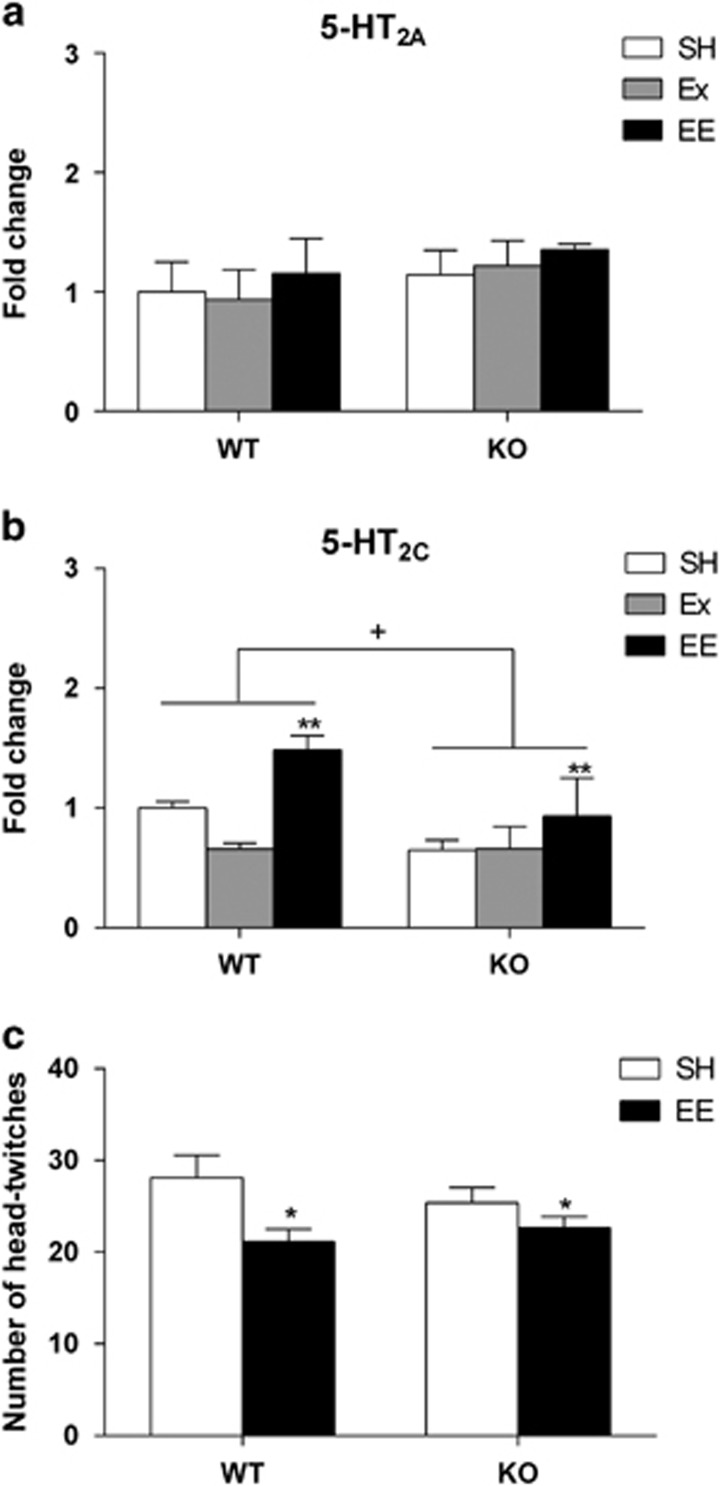
Assessment of 5-HT_2_ gene expression and *in vivo* activity. (**a**) There was no effect of treatment or genotype on serotonin 2A receptor (5-HT_2A_) gene expression. (**b**) We found a main effect of genotype (F_1,22_=6.55, ^+^*P*<0.05) and a specific effect of EE (F_2,22_=7.61, *P*<0.01; SH vs EE, **P<0.01) on serotonin 2C receptor (5-HT_2C_) gene expression. (**c**) *In vivo* assessment of 5-HT_2_ receptor function, by measuring the number of head-twitches induced by the 5-HT_2_ receptor agonist DOI (0.3 mg kg^−1^, i.p.). Overall, EE significantly reduces the number of head-twitches regardless of the genotype (F_1,40_=7.09, *P<0.05). Data are expressed as mean±s.e.m.; qPCR, *n*=2-6; DOI, *n*=8–15. EE, environmental enrichment; qPCR, quantitative PCR; SH, standard housing.

We measured hippocampal gene expression of 5-HT_2_ receptors and then *in vivo* activity of that receptor class (Figure 5). We found no effect of either genotype or treatment for 5-HT_2A_ gene expression (Figure 5a). However, we found a main effect of genotype on 5-HT_2C_ receptor gene expression (Figure 5b; F_1,22_=6.55, *P*<0.05) and a specific effect of EE was also revealed (F_2,22_=7.61; SH vs EE, *P*<0.01). Quantifying other relevant serotonergic targets, we also observed an overall effect of both treatments (F_2,21_=8.09; SH vs Ex, *P*<0.05; SH vs EE, *P*<0.01) on 5-HTT gene expression (Supplementary Figure 4A) and lower 5-HT_3_ receptor expression in 5-HT_1A_R KO animals (Supplementary Figure 4C; F_1,22_=5.53, *P*<0.05). The 5-HT_1B_ and 5-HT_7_ mRNA levels were not influenced by either genotype or treatment (Supplementary Figures 4B and D). Due to the specific effect of EE on 5-HT_2C_ receptor gene expression, we only assessed the effect of EE on 5-HT_2_ receptor function. This was done *in vivo*, by measuring the number head-twitches induced by the 5-HT_2_ receptor agonist DOI (Figure 5c). We revealed a modest but significant effect of EE (F_1,40_=7.09, *P*<0.05). Overall, EE decreased 5-HT_2_ receptor function regardless of the genotype.

## Discussion

### Therapeutic dissociation of Ex and EE effects on cognition and anxiety

Using a novel algorithm-based classification of search strategies in the Morris water maze, we show for we believe the first time that Ex enhances learning performance by increasing the odds for mice to select more hippocampal-dependent or ‘allocentric' search strategies. We also found that Ex had a specific beneficial cognitive effect in the 5-HT_1A_R KO mouse, restoring formation of long-term spatial memory. Overall, our data strongly support a cognitive enhancement induced by voluntary physical activity in mice. In contrast with the cognition data, we found that EE (but not Ex) modified anxiety-like behaviours, demonstrating dissociation between improvements in cognition vs innate anxiety (see Supplementary Table 3 for a summary). Strikingly, although EE had no effect on water maze performance, we show that EE reduced anxiety levels in both WT and 5-HT_1A_R KO mice.

We establish for the first time that Ex accelerates the transition of mice towards using an allocentric search strategy during the spatial learning phase of the Morris water maze (that is, training to acquire the hidden platform location). This effect did not manifest as decreased latency to the platform or path length used in previous studies reporting spatial learning enhancement with Ex.^18, 22, 26^ The complex analysis revealing subtle differences in those measures within our various groups is suggestive of impaired learning in enriched and exercising 5-HT_1A_R KO mice on day 3 and 4, respectively. Latency and path length are tightly correlated measures of learning on the water maze.^39^ However, the differences in latency we report here are in conflict with the path length analysis (that is, on day 3 enriched animals had shorter path lengths than SH, yet KOEE mice had longer latencies than WTEE) so we are reticent to draw major conclusions from those data. Instead, we emphasise that all mice had learned the platform location by day 5 and 6 of training because at that stage we demonstrate this expected correlation in all of our animals and this allows us to interpret the probe test results with more confidence. This provides further evidence that these traditional measures of spatial learning can fail to discriminate between mice acquiring the hidden platform location utilising more or less hippocampal-dependent strategies.^24, 42^ Preferentially adopting allocentric over egocentric strategies to find the hidden platform is hypothesised to enhance long-term spatial memory formation on the water maze retention probe.^42^ Despite increasing allocentric strategy selection frequency, in our study Ex did not enhance WT mice spatial memory (that is, memory engram demonstration of the former platform location). The lack of Ex-induced spatial memory enhancement in young adult mice may be explained by a ceiling effect in our water maze protocol, a claim supported by a similar study in young adult mice receiving 4 weeks of access to Ex, demonstrating no spatial memory enhancement.^45^ Using the identical water maze, that study reported a beneficial effect on spatial memory formation in aged mice housed with 6 months of Ex access, detectable due to the lowered ceiling from poor water maze performance in SH aged mice.

The 5-HT_1A_R KO mouse model is characterised by age-dependent impairments in both spatial learning and memory.^10, 11^ We found only spatial memory to be impaired in our study, potentially resulting from each investigation's different protocols and background strains. However, we do extend these previous findings by establishing a novel spatial learning impairment in the 5-HT_1A_R KO animals as they did not transition to selecting allocentric strategies in a similar manner to WT mice. Emotionality-related aspects of cognition might impact on water maze performance. During spatial learning, we report that genotype and treatment had no effect on thigmotaxis on the first day of training (data not shown), a measure of the immediate stress response to the task, as well as on non-spatial (motivational, motoric) factors during cued learning, since both WT and 5-HT_1A_R KO animals performed similarly when using a visible platform; there were also no differences in experimental body weight gain. Taken together, our spatial learning and control data potentially explains the deficit of spatial memory in the 5-HT_1A_R KO found here and in earlier reports.^10^

Ex but not enrichment rescues the 5-HT_1A_R KO spatial memory deficit, demonstrated by quadrant preference restoration only in 5-HT_1A_R KO mice housed with access to Ex. We also report that the time spent in the target quadrant was reduced in 5-HT_1A_R KO animals, but there was no specific effect of Ex in 5-HT_1A_R KO mice on that measure. The precise origin of the spatial memory deficit in the constitutive 5-HT_1A_R KO is unknown. It may result from a combination of the lack of 5-HT_1A_R activity and compensatory changes to network properties in the hippocampus, resulting from its developmental absence.^47^ These changes include increased basal extracellular 5-HT release, changes to glutamatergic and GABAergic signalling,^48, 49^ as well as loss of auto-inhibitory control in the raphe nucleus in response to stress.^50^ We now also report a reduction of hippocampal 5-HT_2C_ and 5-HT_3_ receptor gene expression in 5-HT_1A_R KO animals.

The fact that EE reduced anxiety-like behaviour without having an effect on Morris water maze performance suggests dissociation between cognitive function vs anxiety levels. It has been proposed that the hippocampal architecture allows it to function as a ‘comparator-behavioural inhibition system'.^24^ Thus it may discriminate between ambiguous and overlapping memories during the water maze but can serve the same function in discrimination between competing behavioural goals on the EPM or novelty-suppressed feeding test (NSFT) (that is, approach or avoid conflict with the open arms/arena centre containing the food pellet). Using the same home-cage EE paradigm without running wheels, as used in the present study, we recently published that early EE has beneficial effects on the anxiety-like behaviour in a mouse model of neurodegenerative disorder with serotonergic dysfunction.^51, 52^ Here, we extend those findings in adult mice to include a beneficial effect in a mouse model of anxiety disorder whose phenotype is developmental in origin.^47^ Selective serotonin reuptake inhibitors (SSRIs), such as fluoxetine, are known to alter innate anxiety on the both the EPM and NSFT (for which the behavioural effect is proposed to be dependent on increased adult hippocampal neurogenesis). However, the behavioural effects of EE (with provision of a running wheel) on the EPM and NSFT are not neurogenesis dependent;^20^ furthermore, the same EE paradigm is still anxiolytic on the EPM with increases to cell proliferation occurring.^53^ We observed no genotype effect on NSFT in our preliminary work (data not shown), but our EPM data, and the conclusions we derive from those results, is in agreement with this literature.

The 5-HT_1A_R has the peculiarity to be expressed at both the pre- and post-synaptic levels.^4^ Unfortunately, constitutive 5-HT_1A_R KO mice do not allow for definitive conclusions about 5-HT_1A_ auto- vs hetero-receptor's relative contributions to cognition or emotion regulation. In adult mice, inducing suppression of 5-HT_1A_ auto-receptors in the raphe using a siRNA or a transgenic approach does not alter innate anxiety.^54, 55, 56^ During development, conflicting results from 5-HT_1A_R auto- or hetero-receptor restoration and suppression point to a combination of raphe and forebrain 5-HT_1A_R signalling changes during development that generate the 5-HT_1A_R KO anxiety phenotype.^38, 47, 55, 57, 58^ EE may be acting directly on raphe signalling or in the forebrain to alter the innate anxiety in both genotypes, although our data indicate the anxiolytic effect of EE is likely independent of 5-HT_1A_R activity. In humans, pre- vs post-synaptic alterations in 5-HT_1A_R are not detectable due to PET imaging limitations.^59^ However, the functional C(-1019)G polymorphism in the 5-HT_1A_R gene has a synergistic effect on 5-HT neurotransmission during development by opposing regulation of auto- and hetero-receptor expression.^5^ Importantly, this polymorphism also determines the responsiveness to SSRIs in patients with anxiety disorders. Since the anxiolytic effect of enrichment in this study does not require 5-HT_1A_R activity, analogous treatment like cognitive-behavioural therapy may be of specific use in SSRI nonresponsive patients.

### Potential mechanisms for the behavioural results

We found that Ex increased cell survival and BDNF levels in both genotypes, but the functional relevance of these increases to optimal (WT) vs suboptimal (KO) cognition is not equivalent. Enhanced hippocampal neurogenesis improves pattern separation, which may increase discrimination between more ambiguous cues.^24^ We hypothesise that this discrimination is impaired in the 5-HT_1A_R KO and that in our study increases in cell survival may restore spatial memory formation through this mechanism. BDNF may also change hippocampal network properties directly, through morphological or electrophysiological changes of existing granule cells. BDNF thus may potentially remedy cognition in the 5-HT_1A_R KO animal independently of hippocampal cellular plasticity changes by rescuing the dysfunction from loss of 5-HT_1A_R activity directly.^33^ Ultimately, in either case, encoding a better allocentric map to the hidden platform location may be the mechanism through which Ex rectifies the 5-HT_1A_R KO cognitive impairment. Interestingly, this learning impairment in the 5-HT_1A_R KO cannot be explained by baseline genotype differences in adult-born cell proliferation, survival or mBDNF and remains as an interesting avenue for future research. We also corroborate in WT mice that EE alters innate anxiety without increasing adult-born cell survival ^20^ and demonstrate that EE improved anxiety-like behaviour in the 5-HT_1A_R KO independently of BDNF levels. Elevated hippocampal BDNF has been found to be anxiolytic but we do not find that effect in exercising mice.^60^

EE reduced anxiety levels in both WT and 5-HT_1A_R KO mice, demonstrating that anxiolytic-like effects of enrichment may be mediated via 5-HT_1A_R independent mechanisms. Our data suggested a specific effect of EE on hippocampal 5-HT_2_ receptors. Since Dunnett's pair-wise comparisons revealed a main effect of only enrichment on 5-HT_2C_R but not 5-HT_2A_R gene expression, we chose to investigate this effect *in vivo* using the number of head-twitches induced by the 5-HT_2_ receptor agonist, DOI. We report that EE modestly decreased the head-twitches by a magnitude that seemed to parallel the increase in 5-HT_2C_ transcription produced by EE. There is evidence that 5-HT_2C_ receptor activation can suppress head-twitch behaviour,^61, 62^ providing a potential explanation for the *in vivo* effect of EE. Taken together, we hypothesise that increased hippocampal 5-HT_2C_R transcription through EE reduces the magnitude of the *in vivo* DOI-induced head-twitch response. Beyond the hippocampus, interactions between 5-HT_2A_R and 5-HT_2C_R are more controversial, with proposed conflicting responses in GABAergic inhibitory control of prefrontal cortex pyramidal cells, as well as opposing control of cortical dopamine efflux.^63, 64^ Reducing cortical 5-HT_2A_ but not 5-HT_2C_ receptor activity has been established as anxiolytic, with the constitutive genetic deletion of 5-HT_2A_R resulting in anxiolytic behaviour that was normalised with cortex specific restoration of 5-HT_2A_R activity.^65^ Since innate anxiety involves non-hippocampal brain regions, lowered cortical 5-HT_2A_R activity is anxiolytic, and increased hippocampal 5-HT_2C_R activity *in vivo* makes it undetectable, further experiments assessing the effect of EE on 5-HT_2_R gene expression in prefrontal cortex are warranted.

### Role of the 5-HT_1A_R in the molecular and cellular effects of EE and Ex

We demonstrate that the 5-HT_1A_R might play an important role in chronic hippocampal cell proliferation increases caused by EE, but seems to not be required for Ex-induced increases in cell survival. It was initially assumed that running increased hippocampal cell proliferation and enrichment increased survival of newborn neurons as separate processes.^18, 21^ However, there is evidence challenging that simplistic cause–effect relationship. Ex also enhances cell survival^66^ while chronic EE (albeit with the provision of a running wheel), can enhance hippocampal cell proliferation.^67^ The suggestions of these latter findings are supported by our observations in WT animals. Recent dissociation studies interrogating the individual and combinational effect of distinct components of EE protocols all identified Ex as the key neurogenic stimulus.^25, 26, 27^ Here, we report Ex-induced increases in adult-born cell survival (two to threefold) and BDNF, which are consistent with these studies. A recent report using a 5-HT_3_R KO identified the receptor as critical for Ex-induced neurogenesis,^31^ however, we do not extend those findings to adult-born cell survival through the 5-HT_1A_R. Furthermore, there is growing evidence suggesting that our reported dissociation in hippocampal function may occur along its dorsal–ventral axis with cognition processed dorsally and anxiety ventrally.^68^ More controversially, differential activity-dependent regulation of some aspects of neurogenesis is also proposed to underpin these distinct behavioural effects along this axis.^53, 67^ However, methodological discrepancies have led to inconsistencies in studies reporting the effects of environmental manipulations on dorsal vs ventral hippocampal neurogenesis.^69^ Acknowledging we could only partially sample the ventral hippocampus also faced with these constraints, we segregated our BrdU and Ki67 coronal sections along this axis. This approach revealed no regional differences in the Ex effect on cell survival or proliferation and a clear Ex effect on cell survival in the ventral hippocampus, suggesting these increases are not contributing to innate anxiety changes in our study. Using sagittal sections to robustly delineate the hippocampal dorsal–ventral axis, dorsal-specific neurogenesis increases and proliferation increases in both regions were reported using an EE protocol with an Ex wheel.^67^ A future direction could use this method to confirm the cell survival effect of Ex alone in the ventral hippocampus.

The EE-induced increase of hippocampal cell proliferation found in WT mice was not observed in 5-HT_1A_R KO animals. Our results suggest a crucial role for intact serotonergic signalling in mediating the proliferative effect of EE. Previous research in the 5-HT_1A_R KO exploring the signalling behind fluoxetine-induced increases in neurogenesis found it essential for this process but dependent on background strain.^36, 37^ New evidence demonstrating that restoration of dentate gyrus 5-HT_1A_R activity in the 5-HT_1A_R KO on a mixed background is sufficient to restore fluoxetine-induced cell proliferation increases, confirms and extends these findings.^38^ Consistent with these findings, we report no baseline cell proliferation or survival differences between SH WT and 5-HT_1A_R KO animals. Chronic increases in cell proliferation are associated with an anxiolytic response on the EPM in both enriched and fluoxetine-treated mice.^38, 53^ Despite reporting proliferation changes in enriched WT but not 5-HT_1A_R KO mice, the anxiolytic enrichment effect in both genotypes suggests innate anxiety changes are not dependent on the activity-dependent changes to proliferation. Thus, the functional relevance of chronic cell proliferation changes in EE constitutes a promising avenue for future research.

## Conclusions

The causal relationship between anxiety and cognition is still unclear, however, we provide clinically relevant evidence here of a dissociation between improvements in those symptoms. In summary, we have demonstrated Ex-induced restoration of a spatial memory deficit in 5-HT_1A_R KO mice, independent of change in affective dysfunction. Overall, the 5-HT_1A_R does not seem to be critical for those behavioural effects to occur. Notably, Ex facilitated the increased use of allocentric search strategies during a spatial learning task coinciding with increased BDNF levels and adult-born cell survival in the hippocampus. In the case of the 5-HT_1A_R KO mice, these effects of Ex coincide with the restoration of the spatial memory deficit. EE alters the affective dysfunction independent of increased adult-born cell survival or BDNF elevation. Furthermore, our findings suggest that the 5-HT_1A_R is not critical for Ex-induced changes in adult-born cell survival, but does potentially mediate the effect of EE on cell proliferation. In humans, functional polymorphisms in the 5-HT_1A_R gene are associated with deficits in cognitive processing and anxiety disorders, as well as insensitivity to 5-HT_1A_R-targeted pharmacology. Here, we demonstrate in mice therapeutically dissociable environmental effects on behaviour that do not require correct 5-HT_1A_R signalling. Thus, we advocate that environmental manipulations, such as Ex or cognitive-behavioural therapy, known to increase efficacy in the treatment of anxiety disorder, may have greater clinical impact if used in tandem to address cognition and affective dysfunction independently.
