# Supplementary material for: Dissociating the therapeutic effects of environmental enrichment and exercise in a mouse model of anxiety with cognitive impairment
Source: Transl Psychiatry. 2016 Apr 26;6(4):e794–. doi: 10.1038/tp.2016.52 (PMC4872410; doi:10.1038/tp.2016.52)
Supplement: Supplementary Information [file tp201652x1.pdf]

Supplementary Table 1 Statistical analysis for all parameters measured using a 2way ANOVA in the study.

Test	Parameter	Fig	ANOVA	F value	P value	Dunnet's	Adj. P value
MWM	Time in target quadrant	1F	Interaction	F (2, 60) = 0.50	P = 0.61	SH vs Ex	N/A
			Genotype	F (1, 60) = 5.59	P = 0.02	SH vs EE	N/A
			Treatment	F (2, 60) = 1.40	P = 0.25		
MWM	Latency to platform	S2C	Interaction	F (2, 60) = 0.081	P = 0.92	SH vs Ex	N/A
	(probe)		Genotype	F (1, 60) = 3.72	P = 0.058	SH vs EE	N/A
			Treatment	F (2, 60) = 0.92	P = 0.41		
MWM	Platform crossings	S2D	Interaction	F (2, 60) = 1.36	P = 0.26	SH vs Ex	N/A
			Genotype	F (1, 60) = 0.042	P = 0.84	SH vs EE	N/A
			Treatment	F (2, 60) = 0.73	P = 0.49		
MWM	Annulus Crossing Index	S2E	Interaction	F (2, 60) = 1.43	P = 0.25	SH vs Ex	N/A
			Genotype	F (1, 60) = 0.22	P = 0.64	SH vs EE	N/A
			Treatment	F (2, 60) = 1.09	P = 0.34		
EPM	Time in open arm	2A	Interaction	F (2, 110) = 0.45	P = 0.64	SH vs Ex	0.82
			Genotype	F (1, 110) = 6.28	P = 0.014	SH vs EE	< 0.001
			Treatment	F (2, 110) = 13.65	P < 0.001		
EPM	Distance travelled	2B	Interaction	F (2, 110) = 0.59	P = 0.55	SH vs Ex	< 0.001
			Genotype	F (1, 110) = 9.33	P = 0.0028	SH vs EE	> 1.0
			Treatment	F (2, 110) = 16.43	P < 0.001		
EPM	Distance per open bout	2C	Interaction	F (2, 110) = 0.18	P = 0.84	SH vs Ex	0.13
			Genotype	F (1, 110) = 4.62	P = 0.034	SH vs EE	0.026
			Treatment	F (2, 110) = 7.89	P < 0.001		
BrdU	Hippocampus	3B	Interaction	F (2, 33) = 0.22	P = 0.80	SH vs Ex	< 0.001
			Genotype	F (1, 33) = 3.83	P = 0.059	SH vs EE	0.094
			Treatment	F (2, 33) = 28.27	P < 0.001		
BrdU	GCL volume	3C	Interaction	F (2, 33) = 1.36	P = 0.27	SH vs Ex	< 0.001
			Genotype	F (1, 33) = 2.02	P = 0.16	SH vs EE	0.42
			Treatment	F (2, 33) = 9.66	P = 0.001		
BrdU	Dorsal	S3A	Interaction	F (2, 33) = 0.29	P = 0.75	SH vs Ex	< 0.001
			Genotype	F (1, 33) = 4.90	P = 0.034	SH vs EE	0.068
			Treatment	F (2, 33) = 29.53	P < 0.001		
BrdU	Ventral	S3B	Interaction	F (2, 21) = 0.82	P = 0.45	SH vs Ex	< 0.001
			Genotype	F (1, 21) = 3.01	P = 0.098	SH vs EE	0.59
			Treatment	F (2, 21) = 39.27	P < 0.001		
Ki67	Hippocampus	3E	Interaction	F (2, 29) = 3.99	P = 0.030	WTSH vs WTEx	0.36
			Genotype	F (1, 29) = 2.17	P = 0.15	WTSH vs WTEE	< 0.001
			Treatment	F (2, 29) = 7.98	P = 0.0017	WTSH vs KOSH	0.56
						WTSH vs KOEx	0.96
						WTSH vs KOEE	0.22
Ki67	SGZ volume	3F	Interaction	F (2, 29) = 0.73	P = 0.49	SH vs Ex	0.0034
			Genotype	F (1, 29) = 0.062	P = 0.80	SH vs EE	0.33
			Treatment	F (2, 29) = 5.96	P = 0.0068		

**Test**	**Parameter**	**Fig**	**ANOVA**	**F value**	**P value**	**Dunnet's**	**Adj. P value**
Ki67	Dorsal	S3C	Interaction	F (2, 29) = 3.58	P = 0.041	WTSH vs WTEx	0.36
			Genotype	F (1, 29) = 3.98	P = 0.057	WTSH vs WTEE	0.0018
			Treatment	F (2, 29) = 5.80	P = 0.0076	WTSH vs KOSH	0.78
						WTSH vs KOEx	1
						WTSH vs KOEE	0.49
Ki67	Ventral	S3D	Interaction	F (2,27) = 1.01	P = 0.38	SH vs Ex	N/A
			Genotype	F (1,27) = 0.0011	P = 0.97	SH vs EE	N/A
			Treatment	F (2,27) = 0.34	P = 0.34		
BDNF	proBDNF	4A	Interaction	F (2, 36) = 0.88	P = 0.42	SH vs Ex	N/A
			Genotype	F (1, 36) = 1.36	P = 0.25	SH vs EE	N/A
			Treatment	F (2, 36) = 1.25	P = 0.30		
	mBDNF	4B	Interaction	F (2, 36) = 0.83	P = 0.44	SH vs Ex	0.023
			Genotype	F (1, 36) = 0.36	P = 0.55	SH vs EE	0.67
			Treatment	F (2, 36) = 5.91	P = 0.0060		
qPCR	5-HT2A	5A	Interaction	F (2, 22) = 0.030	P = 0.97	SH vs Ex	N/A
			Genotype	F (1, 22) = 0.78	P = 0.39	SH vs EE	N/A
			Treatment	F (2, 22) = 0.26	P = 0.77		
qPCR	5-HT2C	5B	Interaction	F (2, 22) = 1.89	P = 0.17	SH vs Ex	0.26
			Genotype	F (1, 22) = 6.55	P = 0.018	SH vs EE	0.044
			Treatment	F (2, 22) = 7.61	P = 0.0031		
qPCR	5-HTT	S4A	Interaction	F (2, 21) = 0.20	P = 0.82	SH vs Ex	0.022
			Genotype	F (1, 21) = 0.86	P = 0.36	SH vs EE	0.0028
			Treatment	F (2, 21) = 8.09	P = 0.0025		
qPCR	5-HT1B	S4B	Interaction	F (2, 22) = 3.03	P = 0.069	SH vs Ex	N/A
			Genotype	F (1, 22) = 0.31	P = 0.58	SH vs EE	N/A
			Treatment	F (2, 22) = 1.87	P = 0.18		
qPCR	5-HT3	S4C	Interaction	F (2, 22) = 0.88	P = 0.43	SH vs Ex	N/A
			Genotype	F (1, 22) = 5.53	P = 0.028	SH vs EE	N/A
			Treatment	F (2, 22) = 0.59	P = 0.57		
qPCR	5-HT7	S4D	Interaction	F (2, 22) = 0.020	P = 0.98	SH vs Ex	N/A
			Genotype	F (1, 22) = 2.79	P = 0.11	SH vs EE	N/A
			Treatment	F (2, 22) = 0.47	P = 0.63		
DOI	Head-twitches	5C	Interaction	F (1, 40) = 1.33	P = 0.26	SH vs Ex	N/A
			Genotype	F (1, 40) = 0.11	P = 0.75	SH vs EE	N/A
			Treatment	F (1, 40) = 7.09	P = 0.011		
Weight gain		S1B	Interaction	F (2, 45) = 0.11	P = 0.90	SH vs Ex	N/A
			Genotype	F (2, 45) = 0.72	P = 0.49	SH vs EE	N/A
			Treatment	F (1, 45) = 1.11	P = 0.30		
Distance run		S1C	Interaction	F (2, 110) = 0.39	P = 0.68	SH vs Ex	N/A
			Genotype	F (1, 110) = 1.73	P = 0.19	SH vs EE	N/A
			Treatment	F (2, 110) = 0.74	P = 0.48		

Supplementary Table 2: Statistical analysis for all parameters measured using a 2way repeated measures ANOVA in the study.

**Test**	**Parameter**	**Fig**	**ANOVA**	**F value**	**P value**	**Bonferroni Interaction**			**Adj. P value**
MWM	Latency to platform	2A	Day	F (5, 280) = 88.28	P < 0.001	**Day*Genotype* Treatment**			
	(learning)		Day*Treatment	F (10, 280) = 0.90	P = 0.53	EE	1	WT vs KO	0.02
			Day*Genotype	F (5, 280) = 1.68	P = 0.14	EE	3	WT vs KO	0.049
			Interaction	F (10, 280) = 2.14	P = 0.022	Ex	4	WT vs KO	0.005
			Treatment	F (2, 56) = 0.18	P = 0.83	SH	1	WT vs KO	0.039
			Genotype	F (1, 56) = 5.66	P = 0.021	WT	1	SH vs EE	0.003
			Interaction	F (2, 56) = 0.96	P = 0.39				
MWM	Path length	2B	Day	F (5, 280) = 70.04	P < 0.001				
			Day*Treatment	F (10, 280) = 5.73	P < 0.001	**Day*Genotype**			
			Day*Genotype	F (5, 280) = 2.95	P = 0.019		4	WT vs KO	0.043
			Interaction	F (10, 280) = 1.48	P = 0.15	**Day*Treatment**			
			Treatment	F (2, 56) = 0.45	P = 0.83		1	SH vs EE	0.014
			Genotype	F (1, 56) = 0.69	P = 0.21		3	SH vs EE	0.014
			Interaction	F (2, 56) = 4.4	P = 0.017		3	SH vs Ex	0.049
MWM	Cued learning	S2A	Day	F (1, 63) = 192.77	P < 0.001				
			Day*Treatment	F (2, 63) = 0.10	P = 0.90				
			Day*Genotype	F (1, 63) = 2.47	P = 0.12				
			Interaction	F (2, 63) = 1.35	P = 0.27				
			Treatment	F (2, 63) = 0.33	P = 0.72				
			Genotype	F (1, 63) = 0.22	P = 0.64				
			Interaction	F (2, 63) = 1.01	P = 0.37				
MWM	Velocity	S2B	Day	F (5, 280) = 0.96	P = 0.45				
	(learning)		Day*Treatment	F (10, 280) = 11.84	P = 0.028	**Day*Treatment**			
			Day*Genotype	F (5, 280) = 2.04	P = 0.089		1	SH vs Ex	0.003
			Interaction	F (10, 280) = 1.77	P = 0.066		1	SH vs EE	0.001
			Treatment	F (2, 56) = 40.10	P < 0.001		2	SH vs Ex	< 0.001
			Genotype	F (1, 56) = 0.18	P = 0.67		3	SH vs Ex	0.005
			Interaction	F (2, 56) = 0.13	P = 0.88		3	SH vs EE	0.007
							4	SH vs Ex	< 0.001
							5	SH vs Ex	< 0.001
							6	SH vs Ex	< 0.001

Supplementary Table 3: A summary of the key results of the study. The behavioural results are listed first followed by the effects on neurogenesis and BDNF. The table is organised to show the genotype effect as well as the effect of both enrichment (EE) and Exercise (Ex). ↓ denotes a significant decrease; ↑ denotes a significantly significant increase; and Ø denotes no statistically significant effect. MWM- Morris water maze; EPM- Elevated plus Maze; mBDNF- mature brain derived neurotrophic factor; 5-HTT- serotonin transporter

Test parameter	Genotype	Ex	EE
Allocentric search strategy selection (MWM)	↓	↑	Ø
Quadrant preference (MWM)	KOSH ↓	KOEx Ø	KOEE ↓
Time in open arm (EPM)	↓	Ø	↑
Distance per open arm bout (EPM)	↓	Ø	↑
Adult-born cell survival	Ø	↑	Ø
Adult-born cell proliferation	Ø	Ø	WTEE ↑
mBDNF level	Ø	↑	Ø
$5\text{-}HT_{2C}$ gene expression	↓	Ø	↑
DOI induced head twitches	Ø	N/A	↓
5-HTT gene expression	Ø	↑	↑
$5\text{-}HT_3$ gene expression	↓	Ø	Ø

SUPPLEMENTARY INFORMATION

Animals and Housing

5-HT1AR knock-out (KO) mice and WT littermates were genotyped by polymerase chain reaction (PCR) using the following primers: CCA ACT ATC TCA GCT CCT T, GCT CCC TTC TTT TCC ACC TTC T, GCC TTC TAT CGC CTT CTT GAC G.

Running distance

Briefly, upon completion of the Morris water maze (MWM), exercised mice were single housed for several days in automated running wheel chambers **(**30 cm x 19 cm x 11 cm; 12 cm diameter wheel**)** to assess any genotype differences in the distance run per night. Activity monitor software (Lafayette Instrument, Lafayette, Indiana, USA) was used to assess the number of wheel revolutions in 5 min bins for the entire 24 h period and converted that into a distance run in Km.

MWM cued learning

A separate cohort of experimentally naïve mice of both genotypes and in each treatment condition were used to control for the ability of mice in each group to learn to swim to a visible goal. This allowed confirmation that the learning impairment in the KO mice was not driven by non-spatial (e.g., motivational, motoric) factors. In this paradigm, a red funnel 10 cm tall was inserted in the middle of the platform and all other visual cues in the room were hidden beneath white sheets. Mice were trained from four novel start positions to find four randomised platform locations. All other elements of the training occurred as described in the methods section.

Dorsal-ventral segregation of hippocampal cell proliferation and survival results

The dorsal and ventral aspect of the hippocampus was delineated according to previous published work using coronal sections (Xia et al., Neurosci Lett. 2012 Jul 11;521(1):20-5). Briefly, a hippocampal section was determined to be dorsal if it was between Bregma -1.22mm and -2.06mm. The very limited sections posterior to Bregma -2.3mm were included as parts of the ventral hippocampus.
