# Supplementary figures and images for: Dissociating the therapeutic effects of environmental enrichment and exercise in a mouse model of anxiety with cognitive impairment
Source: Transl Psychiatry. 2016 Apr 26;6(4):e794–. doi: 10.1038/tp.2016.52 (PMC4872410; doi:10.1038/tp.2016.52)

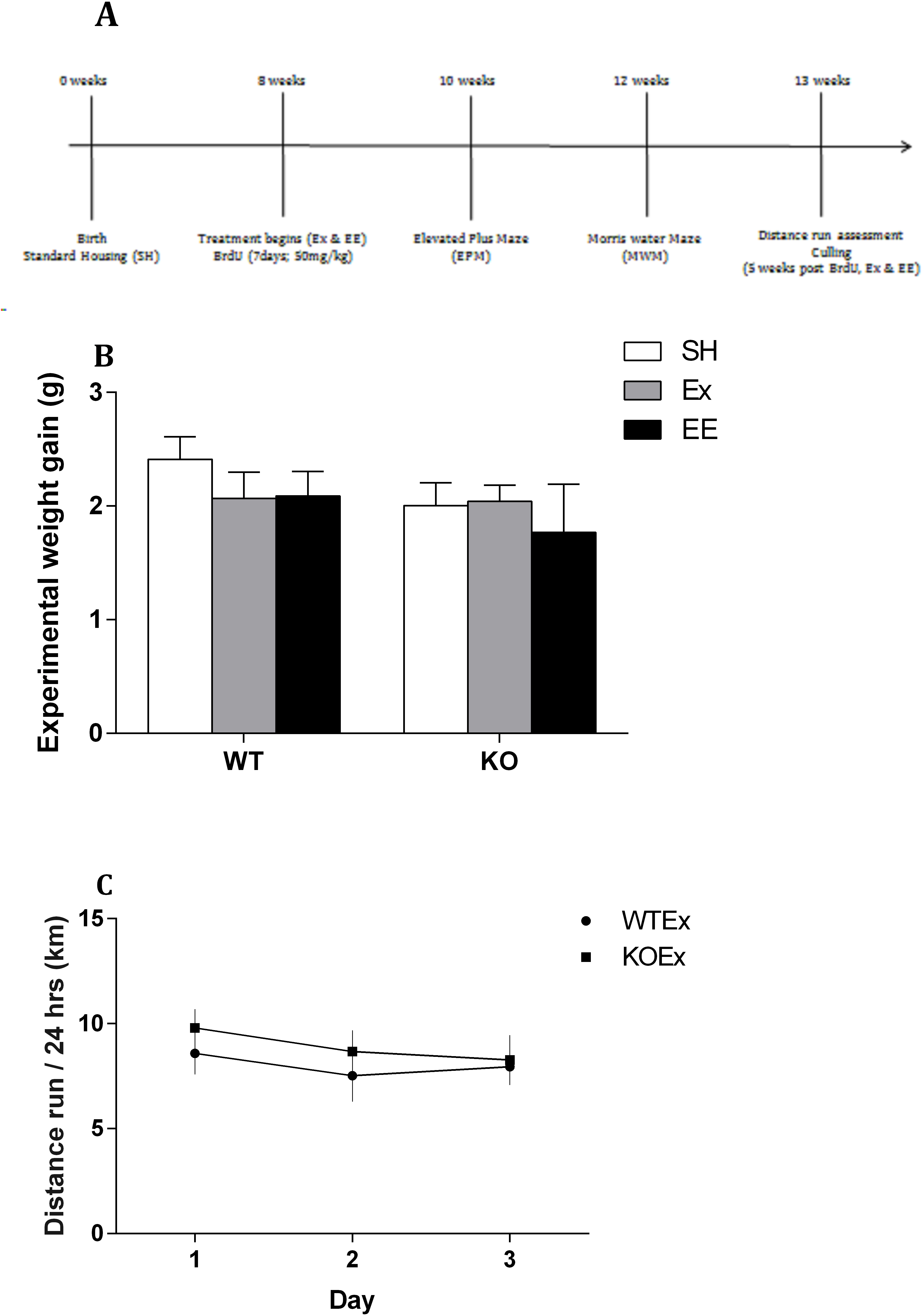

Supplement: Supplementary Information [file tp201652x2.tif]

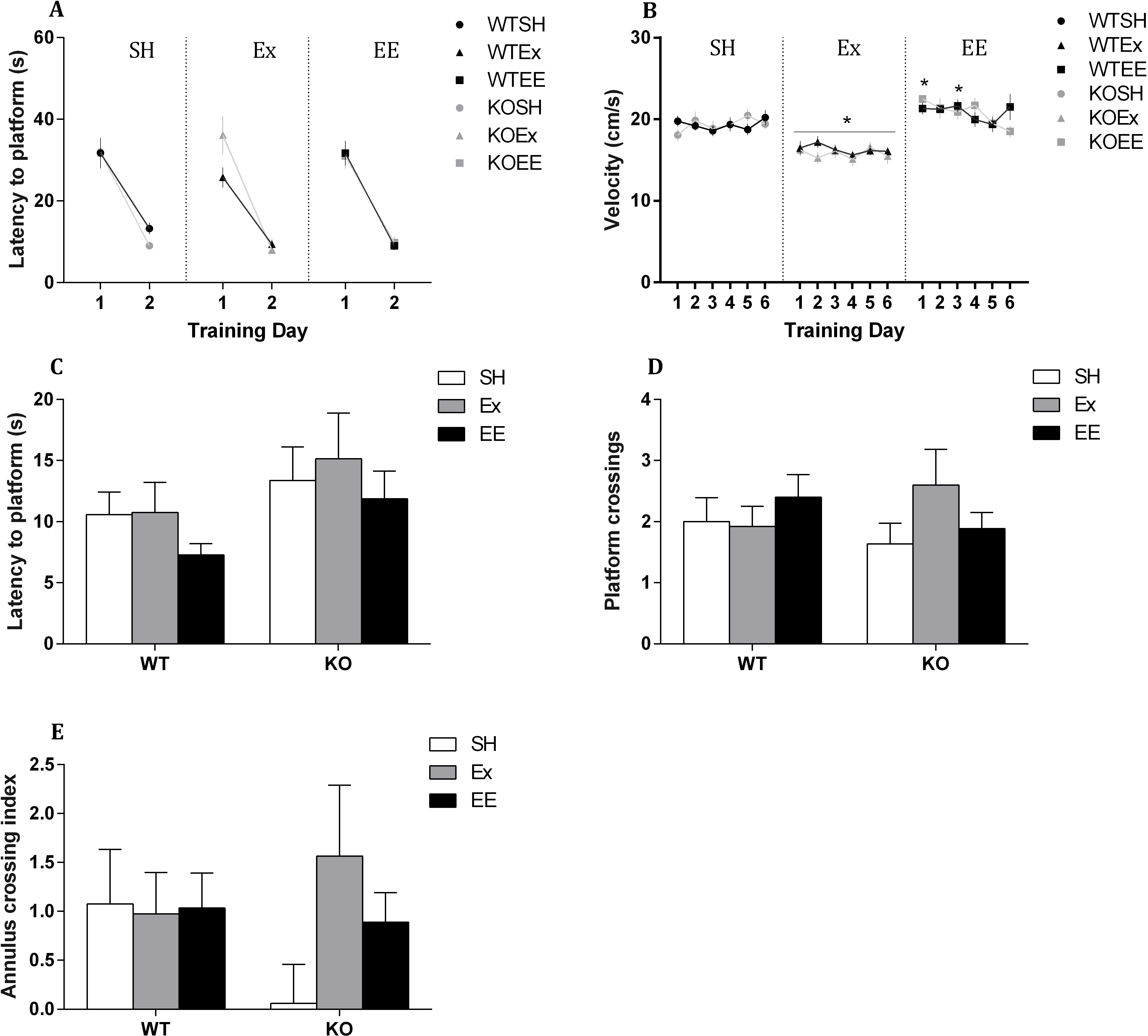

Supplement: Supplementary Information [file tp201652x3.tif]

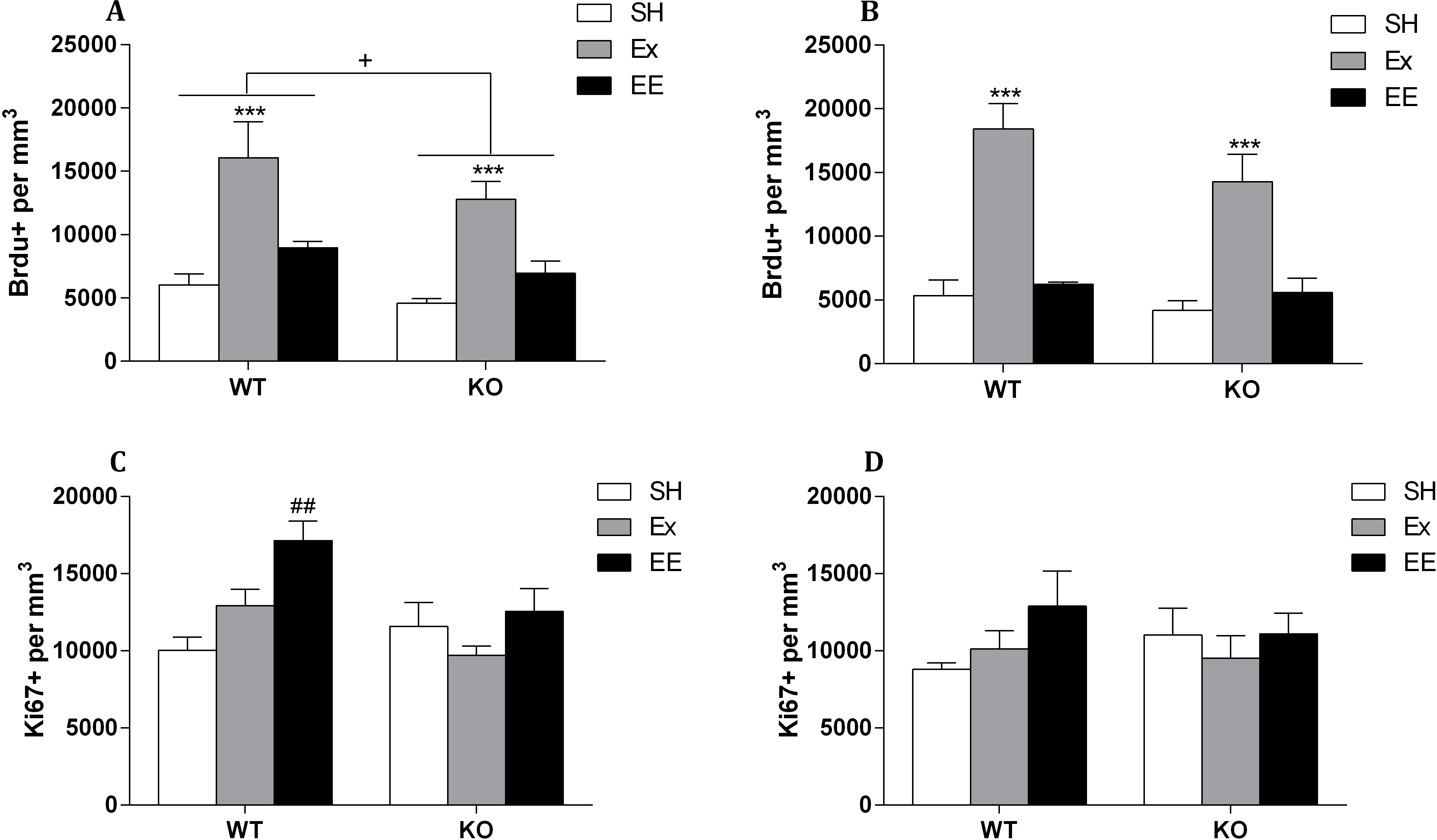

Supplement: Supplementary Information [file tp201652x4.tif]

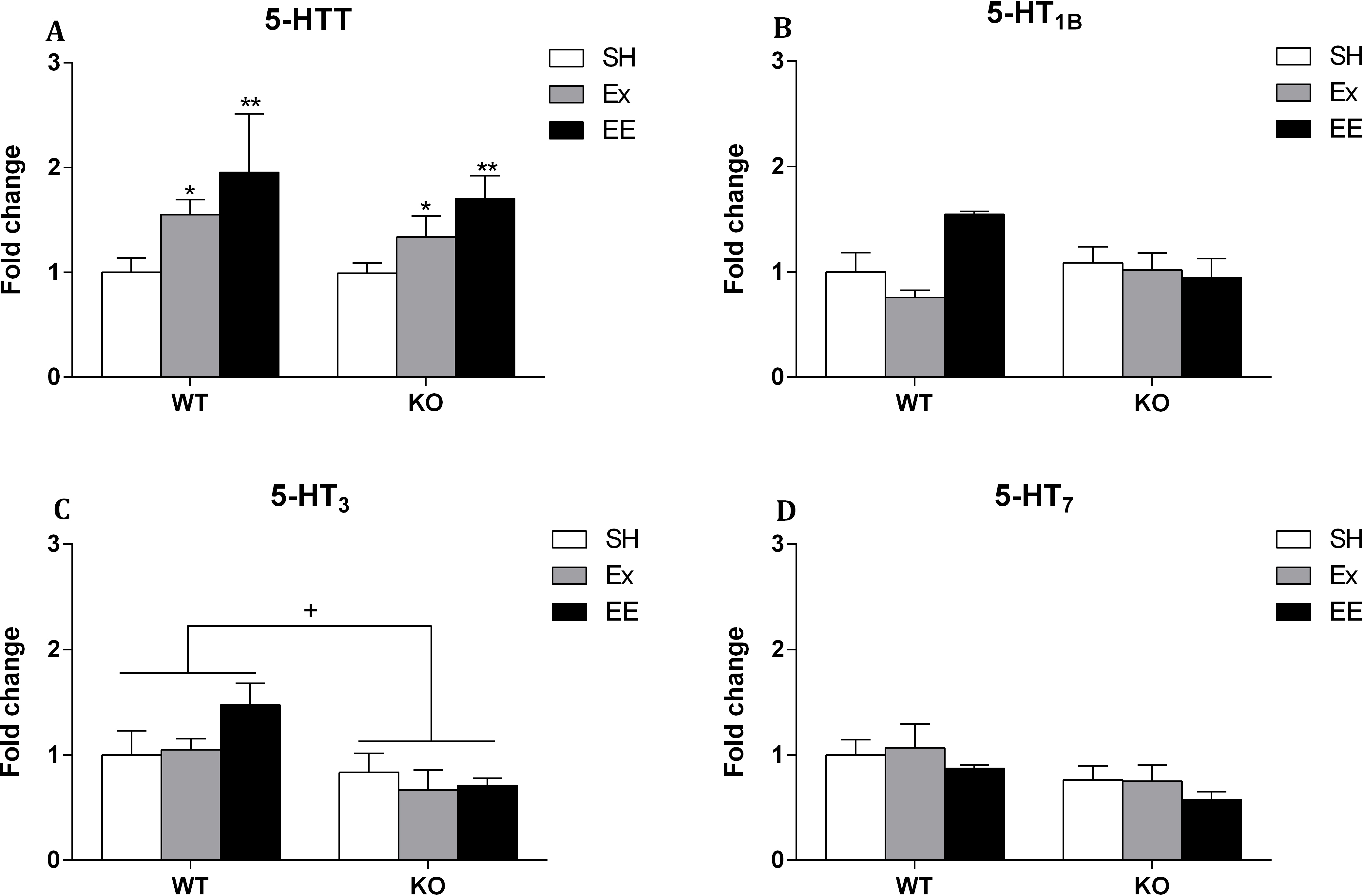

Supplement: Supplementary Information [file tp201652x5.tif]
